# Long non-coding RNA LINC01133 represses KLF2, P21 and E-cadherin transcription through binding with EZH2, LSD1 in non small cell lung cancer

**DOI:** 10.18632/oncotarget.7077

**Published:** 2016-01-30

**Authors:** Chongshuang Zang, Feng-qi Nie, Qian Wang, Ming Sun, Wei Li, Jing He, Meiling Zhang, Kai-hua Lu

**Affiliations:** ^1^ Department of Oncology, First Affiliated Hospital, Nanjing Medical University, Nanjing, People's Republic of China; ^2^ Department of Oncology, Second Affiliated Hospital, Nanjing Medical University, Nanjing, People's Republic of China; ^3^ Department of Biochemistry and Molecular Biology, Nanjing Medical University, Nanjing, People's Republic of China

**Keywords:** NSCLC, lncRNA, EZH2, LSD1, proliferation

## Abstract

Long non-coding RNAs are emerging as crucial regulators and prognostic markers in multiple cancers including non small cell lung cancer (NSCLC). In this study, we screened LINCO1133 as a new candidate lncRNA which promotes NSCLC development and progression, in two independent datasets (GSE18842 and GSE19804) from the Gene Expression Omnibus (GEO). LINC01133 is previously found to be over-expressed in lung squamous cell cancer (LSCC) and knockdown its expression inhibits LSCC cells invasion. However, its' molecular mechanism and downstream targets involving in regulation of cancer cells phenotype is not known. Here, we found that LINC01133 expression is up-regulated in NSCLC tissues, and its' over-expression is associated with patients poor prognosis and short survival time. LINC01133 knockdown decreased NSCLC cells proliferation, migration, invasion and induced cell cycle G1/S phase arrest and cell apoptosis. Mechanistic investigations showed that LINC01133 could interact with EZH2, LSD1 and recruit them to KLF2, P21 or E-cadherin promoter regions to repress their transcription. Furthermore, rescue experiments demonstrated that LINC01133 oncogenic function is partly through regulating KLF2. Lastly, we found that there was negative correlation between LINC01133 and KLF2, P21 or E-cadherin in NSCLC. Overall, our findings illuminate how LINC01133 over-expression confers an oncogenic function in NSCLC that may offer a novel therapy target in this disease.

## INTRODUCTION

Non-small cell lung cancer (NSCLC) accounts for 80% of all lung cancer newly cases, including squamous cell carcinoma (SCC), adenocarcinoma and large cell carcinoma (LCC) [1]. In spite of current advances in the chemotherapy and molecular targeting therapy for NSCLC, the overall 5-year survival rate for NSCLC patients still remains as low as 15% because patients were at advanced stages when diagnosed [2, 3]. Lack of biomarkers for early diagnosis and cancer cells metastasis is still one of the most important reasons challenging NSCLC therapy [4]. Therefore, a greater understanding of the molecular mechanisms involved in the development and progression of NSCLC is essential for the developing of specific diagnostic methods and designing of more individualized and effective therapeutic strategies.

In the past decade, accompany with the fast innovation and development of sequencing technique, many important projects have been achieved such as ENCODE and TCGA, and the importance of long non-coding RNAs (lncRNAs) is emerging from the water [5–7]. lncRNAs are new identified member of non-coding family, which are more than 200 nt in length and often expressed in spatial, temporal and tissue-specific pattern [6, 8–10]. To date, numerous evidence have revealed that lncRNAs involve in a large range of biological processes including X chromosome inactivation, reprogramming stem cell pluripotency, muscle cells differentiation and modulation of cell apoptosis and invasion [11–13]. More importantly, the aberrant lncRNAs expression was found to contribute to many human disease including cancers through the regulation of gene expression by chromatin remodeling, DNA or histone protein modification, and function as sponges for microRNAs [14, 15].

Recently, lncRNAs disorder have been found to participate in NSCLC development and cancer cells metastasis. For example, lncRNA metastasis-associated lung adenocarcinoma transcript 1 (MALAT1) is a highly conserved nuclear lncRNA, which is up-regulated and could be a predictive marker for metastasis development in lung cancer [16]. Increased HNF1A-AS1 promoted lung adenocarcinoma cells proliferation and metastasis through interacting with DNMT1 and repressing E-cadherin expression [17]. In our previous studies, we found that increased HOTAIR and MVIH promoted NSCLC cells invasion and metastasis via regulating HOXA5, MMP2 and MMP9 expression, and lncRNA ANRIL over-expression increased NSCLC cells proliferation by interacting with EZH2 and recruiting it to KLF2 promoter to repress its transcription [18–20]. These data indicated that lncRNAs play important roles in NSCLC pathogenesis, which could provide new insights into the biology of this disease. Hence, more lncRNA need be identified and investigated.

The lncRNA LINC01133, 1154nt in length, is located in chromosome1q23.2. LINC01133 was recently found to be up-regulated in LSCC tissues and its over-expression is associated with LSCC patients shorter survival time, while knockdown of LINCO1133 inhibited invasion ability of LSCC cell [21]. However, the expression pattern, biological function and underlying mechanism in NSCLC is still completely unknown, which need to be investigated. The aim of this study was to investigate misregulated lncRNAs in NSCLC by analyzing microarray data from GEO datasets, and we focus on the highly up-regulated lncRNA LINC01133. Furthermore, loss and gain of function was performed to investigate the contributions of LINC01133 to NSCLC cell phonotype and the potential molecular mechanism was also investigated. The present study will provide new insights into the biological functions of lncRNA LINC01133 as well as its regulatory mechanisms of targets in NSCLC.

## RESULTS

### LINC01133 expression was up-regulated and correlated with poor prognosis of NSCLC

**Figure 1:**
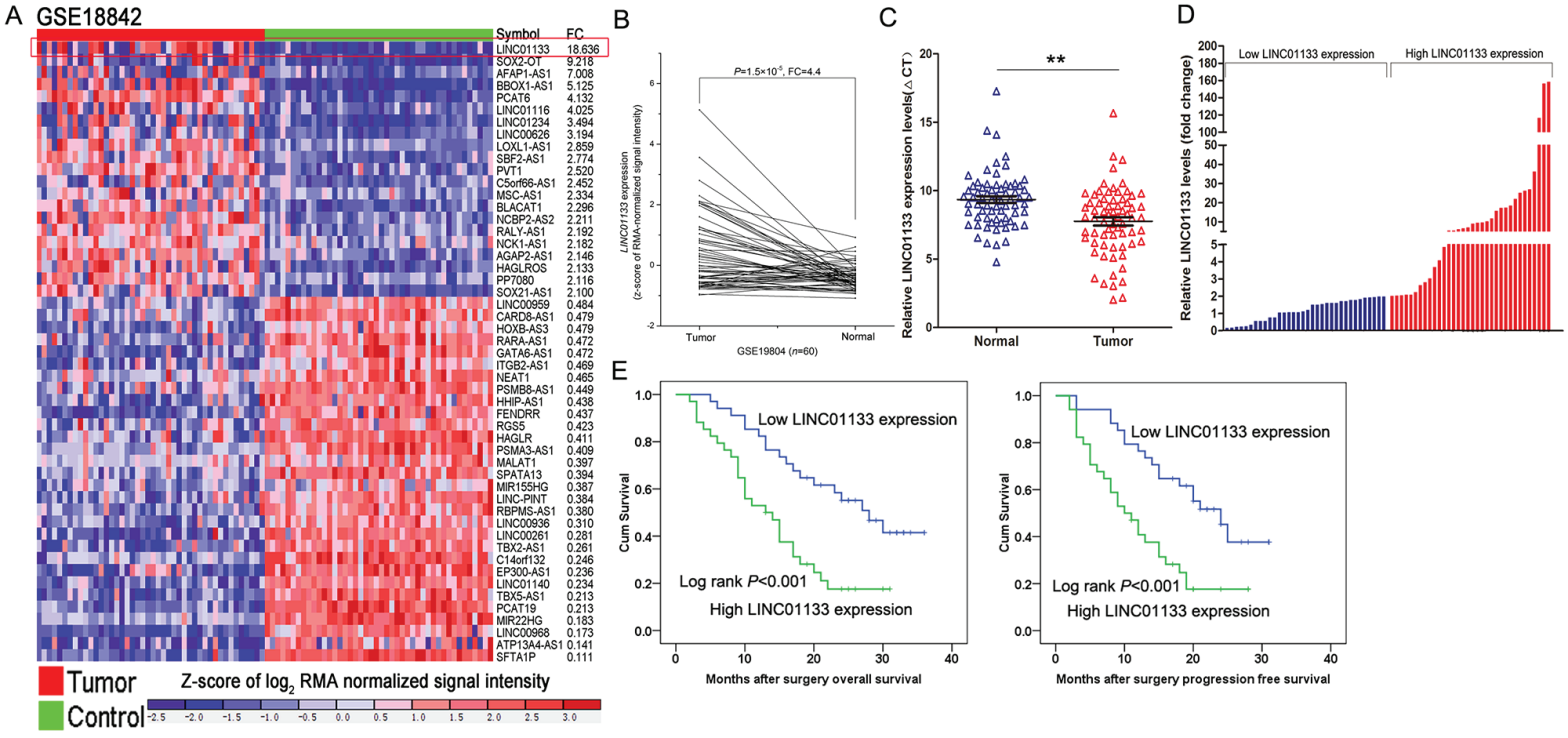
Relative LINC01133 expression in NSCLC tissues and its clinical significance **A, B.** Relative expression of LINCO1133 in NSCLC tissues compared with normal tissue was analyzed by using GEO datasets GSE18842 and GSE19804. **C.** Relative expression of LINCO1133 in NSCLC tissues (*n* = 68) compared with corresponding non-tumor tissues (*n* = 68) was examined by qPCR and normalized to GAPDH expression. Results were presented as the delta CT value. **D.** LINC01133 expression was classified into two groups. **E.** Kaplan–Meier overall survival and disease-free survival curves according to LINC01133 expression levels. *P <0.05, **P <0.01.

To investigate aberrant lncRNAs in NSCLC, we firstly analyzed the microarray data from GEO datasets and found that LINC01133 was the highest up-regulated lncRNA in GSE18842 dataset (Figure 1A). In addition, another independent dataset GSE19804 was used to confirmed the LINC01133 expression, which revealed that LINC01133 also was up-regulated about 4.4 fold (Figure 1B). Furthermore, LINC01133 expression levels were detected in an cohort of 68 pairs NSCLC tissues and adjacent normal tissues by using qPCR. The results showed that LINC01133 expression was significantly up-regulated (Fold change >1.5, *P* <0.01) in 74% (50/68) of cancerous tissues compared with normal tissues (Figure 1C). Increased LINC01133 expression levels in NSCLC were significantly correlated with tumor size (*p* = 0.015), advanced pathological stage (*p* = 0.009) and Lymph node metastasis (*p* = 0.015). However, LINC01133 expression was not associated with other parameters such as gender (*p* = 0.324) and age (*p* = 0.467) in NSCLC (Table 1).

**Table 1:** Correlation between LINC01133 expression and clinicopathological characteristics of NSCLC patients

Characteristics	LINC01133		P Chi-squared test P-value
	High No. cases (34)	Low No. cases (34)	
**Age(years)**			0.467
≤65	15	18	
>65	19	16	
**Gender**			0.324
Male	18	22	
Female	16	12	
**Histological subtype**			0.451
Squamous cell carcinoma	20	23	
Adenocarcinoma	14	11	
**TNM Stage**			0.009*
Ia + Ib	4	14	
IIa + IIb	12	12	
IIIa	18	8	
**Tumor size**			0.015*
≤5cm	13	21	
>5cm	21	13	
**Lymph node metastasis**			0.015
Negative	10	20	
Positive	24	14	
**Smoking History**			0.200
Smokers	20	25	
Never Smokers	14	9	

* Overall *P* < 0.05

Kaplan-Meier survival analysis was conducted to investigate the correlation between LINC01133 expression and NSCLC patients prognosis. According to relative LINC01133 expression in tumor tissues, the 68 NSCLC patients were classified into two groups: the high LINC01133 group (*n* = 34, fold-change ≥ mean ratio); and the low LINC01133 group (*n* = 34, fold-change ≥ mean ratio) (Figure 1D). The overall survival rate over 3 years for the high LINC01133 group was 21.1%, and 41.5% for the low LINC01133 group. Median survival time for the high LINC01133 group was 21months, and 30 months for the low LINC01133 group (Figure 1E). With respect to progression-free survival (PFS), this was 17.6%for the high LINC01133 group, and 37.7% for the low LINC01133 group. Median survival time for the high LINC01133 group was 19 months, and 27 months for the low LINC01133 group (Figure 1F).

### Modulation of LINC01133 expression in NSCLC cells

We next performed qPCR analysis to examine the expression of LINC01133 in 8 human NSCLC cell lines, including both adenocarcinoma and squamous carcinoma subtypes (Supplementary Figure S1A). To investigate the functional effects of LINC01133 in NSCLC cells, we modulated its expression through transfection of LINC01133 siRNA or shRNA to knockdown its expression, and LINC01133 vector to over-express its expression. QPCR analysis of LINC01133 levels was performed 48 h post-transfection, and the results showed that LINC01133 expression was knocked down or over-expressed by si-LINC01133, sh-LINC01133 or pCDNA-LINC01133 transfection when compared with control cells (Supplementary Figure S1B and S1C).

### Knockdown of LINC01133 impaired NSCLC cells proliferation and induced apoptosis

**Figure 2:**
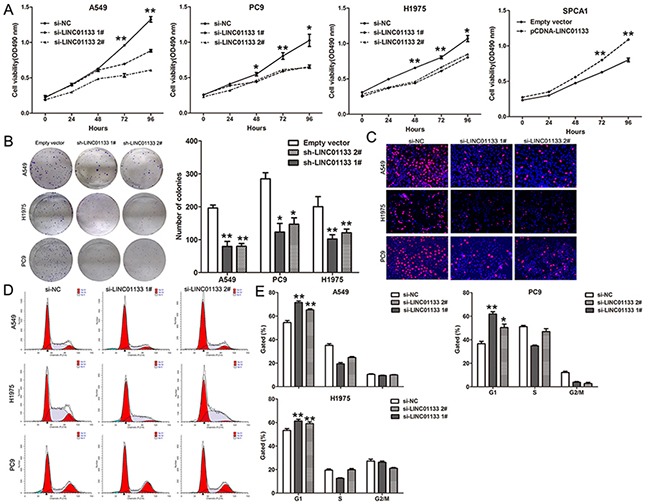
Effects of LINC01133 on NSCLC cell proliferation and cell cycle progression *in vitro* **A.** MTT assays were used to determine the cell viability for si-LINC01133-transfected A549, PC9 and H1975 cells, or pCDNA-LINC01133 transfected SPCA1 cells. Values represented the mean ± s.d. from three independent experiments. **B.** Colony-forming assays were conducted to determine the proliferation of si-LINC01133-transfected A549, PC9 and H1975 cells. **C.** EdU staining assays were conducted to determine the viability of si-LINC01133-transfected A549, PC9 and H1975 cells. **D.** Flow cytometry assays were performed to analysis the cell cycle progression when NSCLC cells transfected with si-LINC01133. The bar chart represented the percentage of cells in G0/G1, S, or G2/M phase, as indicated. All experiments were performed in biological triplicates with three technical replicates.*P < 0.05, **P < 0.01.

To assess the roles of LIN01133 in NSCLC, we performed loss- and gain-of-function assays. MTT assays revealed that cell growth was inhibited in A549, H1975 and PC9 cells transfected with si-LINC0113 compared with controls. In contrast, over-expression of LINC01133 could promote SPCA1 cells (with relative low endogenous LINC01133 expression level) proliferation (Figure 2A). Colony formation assay results revealed that clonogenic survival was inhibited following down-regulation of LINC01133 in A549, H1975 and PC9 cells, while LINC01133 over-expression increased SPCA1 cells clone formation ability (Figure 2B and Supplementary Figure S1D). In addition, EdU staining assays also indicated that LINC01133 knockdown decreased NSCLC cells proliferation, while its over-expression increased NSCLC cells proliferation (Figure 2C).

**Figure 3:**
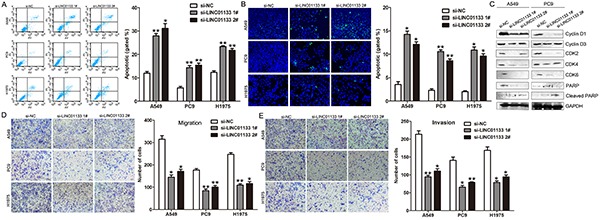
Knockdown of LINC01133 induced NSCLC cell apoptosis and inhibited cell migration and invasion *in vitro* **A.** Flow cytometry assays were performed to analysis the cell apoptotic in si-LINC01133-transfected A549, PC9 and H1975 cells. **B.** Tunel staining assays were performed to analysis the cell apoptotic when knockdown of LINC01133. **C.** Effect of knockdown of LINC01133 on cell cycle and apoptosis related proteins expression. **D, E.** Effect of knockdown of LINC01133 on cell migration and invasion. Data are presented as mean ± SD. *P < 0.05, **P < 0.01.

To further examine whether the effect of LINC01133 on proliferation of NSCLC cells reflected cell cycle arrest, cell cycle progression was analyzed by flow cytometry analysis. The results revealed that A549, H1975 and PC9 cells transfected with si-LINC01133 had an obvious cell cycle arrest at the G1/G0 phase and a decreased G2/S phase (Figure 2D and 2E). To determine whether NSCLC cell proliferation was influenced by cell apoptosis, we performed flow cytometry and Tunel staining analysis. The results showed that NSCLC cells transfected with LINC01133 siRNA showed higher apoptotic rate in comparison with control cells (Figure 3A and 3B). Moreover, some cell cycle and apoptosis related proteins levels were detected, and the results showed that the levels of Cyclin D1, Cyclin D3, CDK2, CDK4, and CDK6 were decreased in LINC01133 knockdown cells, and Cleaved PARP level was significantly increased (Figure 3C). These data indicate that LINC01133 could promote the proliferation phenotype of NSCLC cells.

### Decreased LINC01133 expression inhibits NSCLC cells migration and invasion

As cancer cells migration and invasion is a significant aspect of cancer progression, we investigate the effect of LINC01133 on NSCLC cells migration and invasion by performing transwell assays. The results showed that decreased LINC01133 expression impeded the NSCLC cells migration and invasion compared with controls (Figure 3D and 3E). These results indicated that knockdown of LINC01133 had tumor-suppressive effects that could inhibit migration and invasion in NSCLC cells.

### Down-regulation of LINC01133 inhibits NSCLC cells tumorigenesis *in vivo*

**Figure 4:**
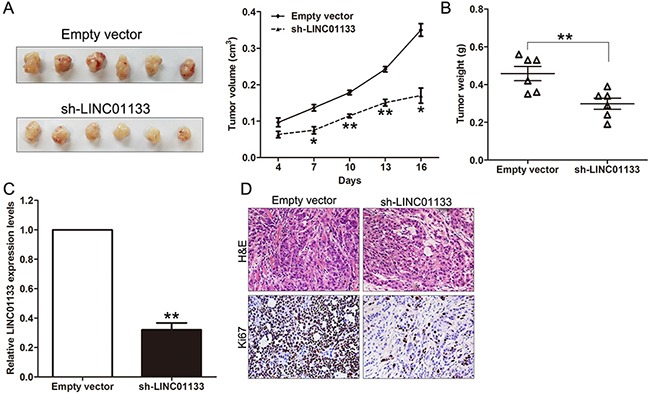
Effect of LINC01133 knockdown on tumor growth *in vivo* **A.** The stable LINC01133 knockdown A549 cells were used for the *in vivo* study. The nude mice carrying tumors from respective groups were shown and tumor growth curves were measured after the injection of A549 cells. Tumor volume was calculated every 3 days. **B.** Tumor weights are represented. **C.** QPCR analysis of LINC01133 expression in tumor tissues formed from A549/sh-LINC01133 and A549/empty vector. **D.** Tumors developed from sh-LINC01133 transfected A549 cells showed lower Ki67 protein levels than tumors developed by control cells. Upper: H & E staining; Lower: immunostaining. *P < 0.05, **P < 0.01.

To explore whether the level of LINC01133 expression could affect NSCLC cells tumorigenesis, A549 cells stably transfected with sh-LINC01133 or empty vector were inoculated into female nude mice. Eighteen days after the injection, the tumors formed in the sh-LINC01133 group were substantially smaller than those in the control group (Figure 4A). Moreover, the tumor weight at the end of the experiment was markedly lower in the sh-LINC01133 group (0.458 ± 0.093 g) compared with the empty vector group (0.298 ± 0.073 g) (Figure 4B). QPCR analysis found that the levels of LINC01133 in tumor tissues formed from sh-LINC01133 cells were lower than in tumors formed in the control group (Figure 4C). Tumors formed from sh-LINC01133 transfected A549 cells exhibited decreased positive for Ki67 than those from control cells (Figure 4D). These findings indicate that knockdown of LINC01133 inhibits tumor growth *in vivo*.

### LINC01133 suppressed KLF2, P21 and E-cadherin transcription by interacting with EZH2 and LSD1

**Figure 5:**
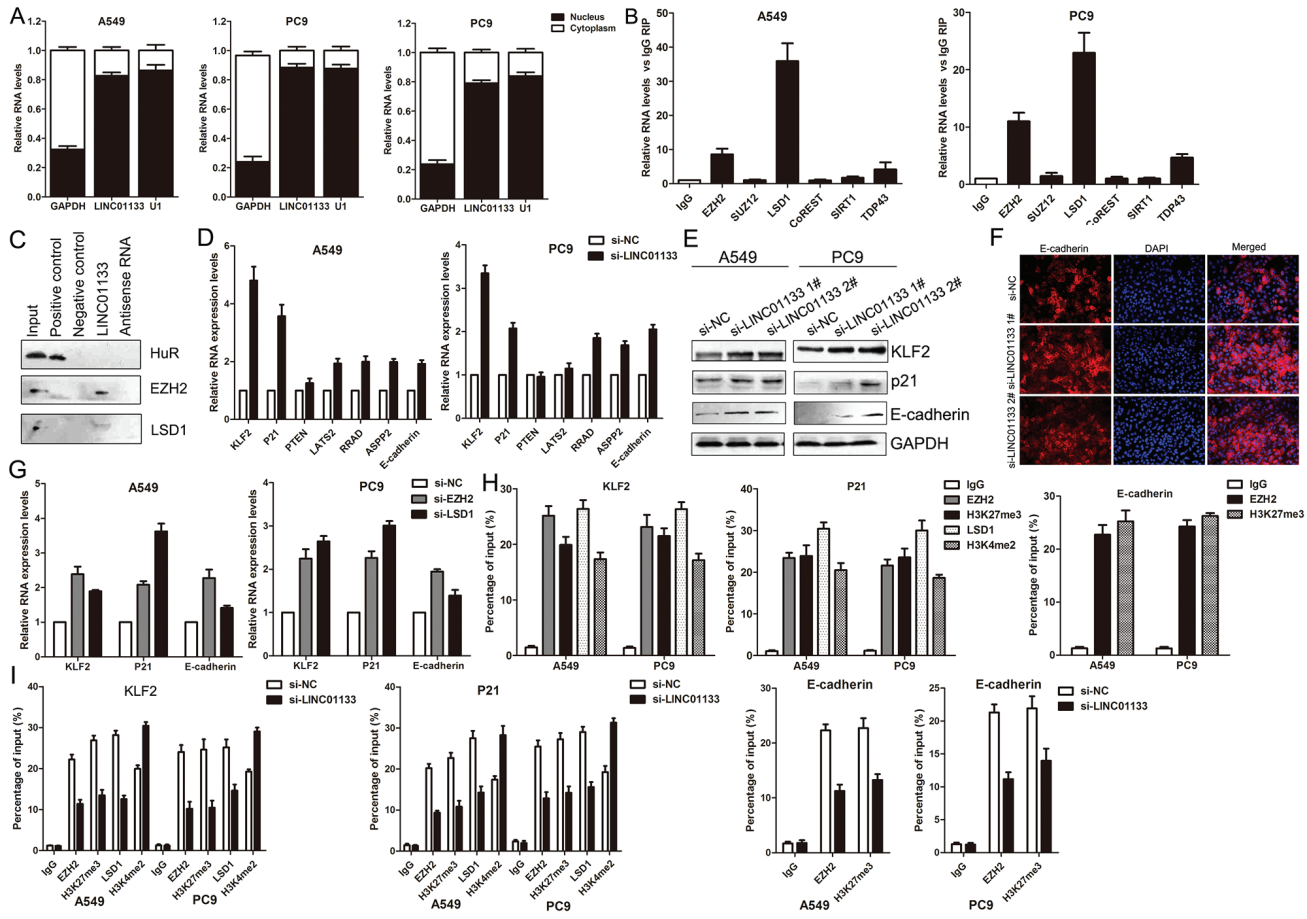
LINC01133 interacted with EZH2 and LSD1, and silence KLF2, P21 and E-cadherin expression **A.** Relative LINC01133 levels in cell cytoplasm or Nucleus of NSCLC cell lines were detected by qPCR. **B.** RNA levels in immunoprecipitates were determined by qPCR. Expression levels of LINC01133 RNA were presented as fold enrichment relative to IgG immunoprecipitates. **C.** Protein levels in immunoprecipitates were determined by western blot. Expression levels of EZH2 and LSD1 protein were presented. **D.** The levels of KLF2, P21, PTEN, LATS2, RRAD, ASPP2 and E-cadherin mRNA were determined by qPCR when knockdown of LINC01133. **E.** The KLF2, P21 and E-cadherin protein levels were determined by western blot in LINC01133 knockdown A549 and PC9 cells. **F.** The E-cadherin protein levels were determined by immunofluorescence analysis in LINC01133 knockdown A549 cells. **G.** The KLF2, P21 and E-cadherin expression levels were determined by qPCR when knockdown of EZH2 or LSD1 in A549 and PC9 cells. **H, I.** ChIP–qPCR of EZH2 and LSD1 occupancy, H3K27me3 and H3K4me2 binding in the KLF2, P21 or E-cadherin promoter in A549 and PC9 cells, and IgG as a negative control. The mean values and s.d. were calculated from triplicates of a representative experiment.

Previously studies have indicated that lncRNAs contribute to cancer cells phenotype through silencing of tumor suppressors or activation of oncogenes via interacting with specific RNA binding proteins. To investigate the potential mechanism of LINC01133 in NSCLC cells, we firstly analysis the distribution of LINC01133 in NSCLC cells and found that LINC01133 mostly located in nucleus (Figure 5A). Then we performed RIP assays to determine the RNA binding proteins (regulate targets at transcriptional levels) which would interact with LINC01133 in NSCLC cells. The results showed that LIN01133 could bind with EZH2 and LSD1 but not other RNA binding proteins in NSCLC cells, and HOTAIR which could also simultaneously bind with EZH2 and LSD1 was used as control ((Figure 5B and Supplementary Figure S1F). In addition, RNA pulldown assays also indicated that LINC01133 could directly binding with EZH2 and LSD1 in A549 cells (Figure 5C).

According to the RIP and RNA pulldown results, we selected some important EZH2 or LSD1 underlying targets and hypothesized that they may also involved in the contributions of LINC01133 to NSCLC development. The results of qPCR showed that KLF2, P21 and E-cadherin expression was increased in A549 and PC9 cells with transfection of si-LINC01133; however, there was no significant difference of other genes when knockdown of LINC01133 expression (Figure 5D). Meanwhile, the western blot and immunofluorescence assays showed the same results (Figure 5E and 5F), which indicated that KLF2, P21 and E-cadherin could be LINC01133 novel targets in NSCLC cells. Furthermore, our qPCR results also showed that inhibition of EZH2 expression led to increased KLF2, P21 and E-cadherin expression and knockdown of LSD1 up-regulated P21 and KLF2 (Figure 5G). To further investigate whether LINC01133 repressed KLF2, P21 and E-cadherin expression through interacting with EZH2 and LSD1, we performed ChIP analysis and the results showed that EZH2 could directly bind to KLF2, P21 and E-cadherin promoter regions and mediate H3K27me3 modification, while LSD1 could bind to P21 and E-cadherin promoter region and mediate H3K4me2 demethylation (Figure 5H). However, knockdown of LINC01133 reduced their binding ability (Figure 5I). These data indicated that LINC01133 contributes to NSCLC cells proliferation and apoptosis partly through repressing KLF2 and P21, while regulates cell migration and invasion via silencing E-cadherin expression in NSCLC cells.

To explore entire repertoire of LINC01133-affected genes in NSCLC, we further performed an RNA Seq analysis after silencing of LINC01133 in A549 cells. We found that 307 genes expression was upregulated (log2 fold change>2), and 149 genes expression was down regulated (Supplementary Figure S2A). Moreover, many of these genes are involved in cell growth, cell proliferation and cell death et.al biological process (Supplementary Figure S2B). Next, three of these upregulated genes (EGR1, ATF3 and DDIT3) expression was conformed in A549 cells after knockdown of LINC01133 (Supplementary Figure S2C). These data indicated that there are also additional LINC01133 target genes that are relevant to the observed biological phenotypes

### Silence of KLF2 is partly involved in the oncogenic function of LINC01133

**Figure 6:**
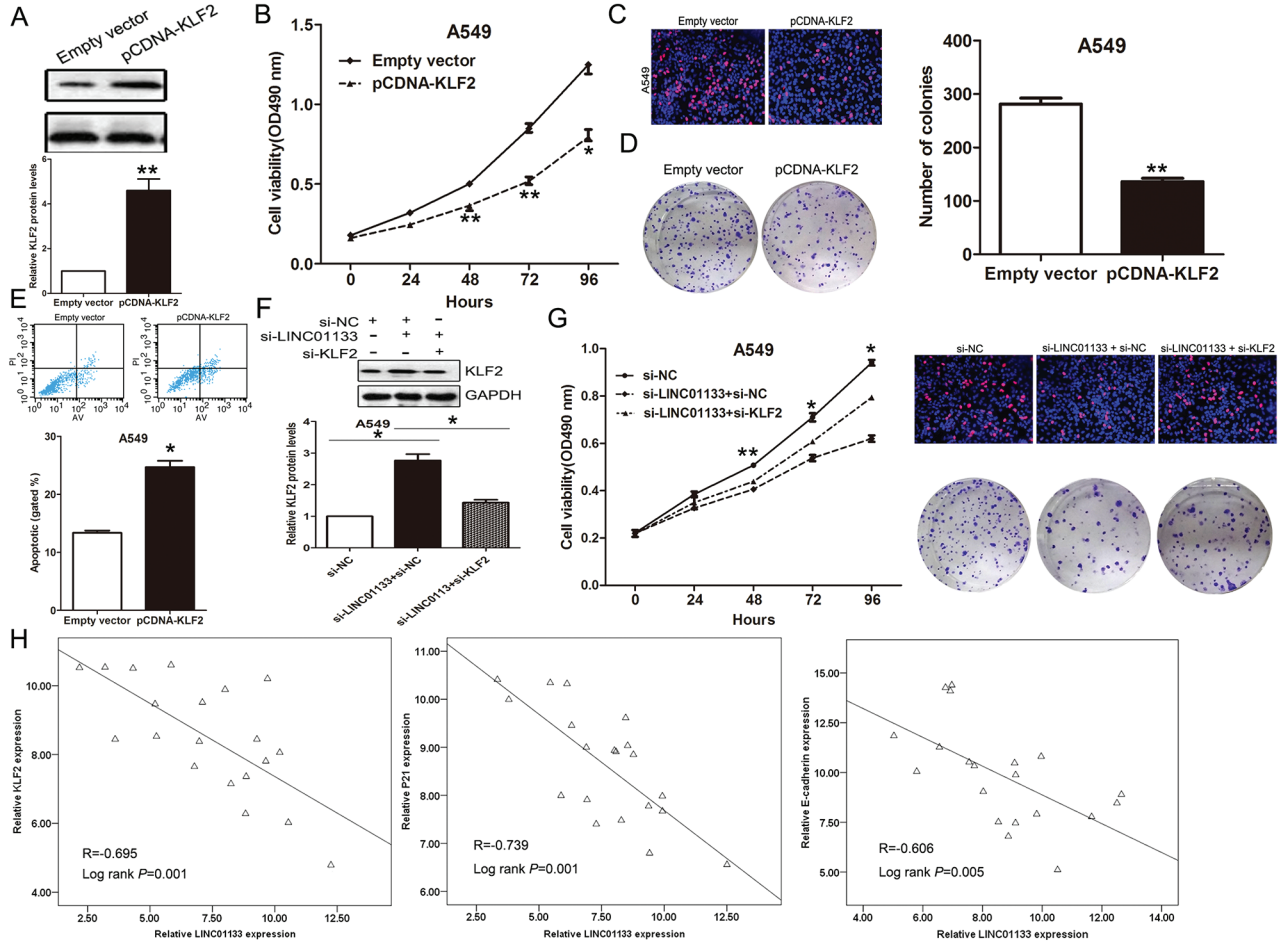
LINC01133 function as oncogene by repressing KLF2 expression in NSCLC cells A549 cells were transfected with pCDNA-KLF2, or co-transfected with si-LINC01133 and si-KLF2. **A.** The protein level of KLF2 was detected by western blot. **B, C, D.** MTT assays, colony-forming and EdU staining assays were used to determine the cell viability. Values represent the mean ± s.d. from three independent experiments. **E.** Apoptosis was determined by flow cytometry. **F.** The protein level of KLF2 was detected by western blot. **G.** MTT assays, colony-forming and Edu staining assays were used to determine the cell viability. **H.** Analysis of the relationship between LINC01133 expression and KLF, P21 or E-cadherin expression levels. *P <0.05 and **P <0.01.

To investigate whether KLF2 is involved in the LINC01133-induced promotion of NSCLC cell proliferation, we performed gain of function assays. As the biological function of KLF2 in PC9 cells has been documented in our previous study, we performed gain of function assays in A549 cells. The results of western blot showed that KLF2 expression was significantly up-regulated in A549 cells transfected with pCDNA-KLF2 compared with control cells (Figure 6A). Meanwhile, MTT, colony formation and EdU assays revealed that over-expression of KLF2 could impair A549 cells proliferation (Figure 6B, 6C and 6D). Moreover, flow cytometry analysis indicated that increased KLF2 expression induced A549 cells apoptosis (Figure 6E). Furthermore, to determine whether LINC01133 regulate NSCLC cell proliferation via repressing KLF2 expression, rescue assays were performed. A549 cells were co-transfected with si-LINC01133 and si-KLF2, and this was shown to rescue the increased expression of KLF2 induced by knockdown of LINC01133 (Figure 6F). The results of MTT, colony formation and EdU assays results indicated that co-transfection could partially rescue si-LINC01133-impaired proliferation in A549 cells (Figure 6G). Further analysis revealed that LINC01133 expression is inversely correlated with KLF2, P21 and E-cadherin level in 20 paired NSCLC tissues (Figure 6H). These data indicate that LINC01133 regulates NSCLC cell proliferation partly through the down-regulation of KLF2 expression.

## DISCUSSION

Recently, more and more studies have revealed that lncRNAs play important roles in multiple cancers development and progression, including NSCLC [22–24]. For example, lncRNA XIST promotes human glioblastoma stem cells proliferation, migration and invasion by sponging miR-152[25]. In addition, lncRNA BANCR exerts tumor-suppressive functions in NSCLC cells, while HOTAIR, MALAT1, LUADT1 and AFAP1-AS1 et.al exert oncogenic function in NSCLC [18, 26–29]. Our previous studies also revealed that lncRNA ANRIL and MVIH function as oncogenes in NSCLC cells, while MEG3 exerts tumor-suppressive function [30]. In this study, our screened another lncRNA LINC01133 that is significantly up-regulated in NSCLC tissues by analyzing GEO datasets, and increased LINC01133 expression may be critically involved in the NSCLC development. Meanwhile, LINC01133 expression also is up-regulated in LSCC (one major histological type of NSCLC), but not in Lung adenocarcinoma (LAD); however, we found that LINC01133 expression is overexpressed in both NSCLC types and correlated with patients poor prognosis, which may due to the specific expression pattern of lncRNA in different population. More important, there is no study that revealed the molecular mechanism and downstream targets of LINC01133 until now. Here, we demonstrated that knockdown of LINC01133 expression exerted tumor-suppressive effects through impairing the cell proliferation, migration and invasion, and inducing cell apoptosis. Furthermore, this study provided evidence for the first time that LINC01133 exerted oncogenic functions in human NSCLC cells by interacting with EZH2 and LSD1, and repressing KLF2, P21 and E-cadherin expression.

Generally, lncRNAs regulate downstream targets through interacting with specific RNA binding proteins and leading to inactivation or activation of gene expression via chromosome reprogramming, DNA methylation, histone protein modification or RNA decay [31, 32]. Our data revealed that LINC01133 could simultaneously interact with EZH2 and LSD1 to repress KLF2, P21 and E-cadherin expression in NSCLC cells. EZH2 is an core subunit of PRC2 complex that could catalyze the trimethylation of lysine residue 27 of histone 3 (H3K27me3), which is over-expressed in multiple cancers [33]. Highly EZH2 protein expression level is also associated with the early pathogenesis and prognosis of NSCLC patients, and promotes tumor progression via regulating VEGF-A/AKT signaling [34]. In addition, LSD1, one of the enzymatic core of the REST repressor, is the first identified histone demethylase, specifically H3K4me1/2 demethylase [35]. Recent study also revealed that LSD1 is over-expressed in NSCLC and increased LSD1 promoted cell proliferation, migration and invasion [36]. The present study demonstrated that LINC01133 could bind with EZH2 and LSD1, and recruit them to KLF2, P21 or E-cadherin promoter regions to repress their expression in NSCLC cells. These data indicated that LINC01133 may play critical roles in EZH2 and LSD1 mediated repression of tumor suppressors in NSCLC cells.

KLF2, an member of KLF family with Cys2/His2 zinc-finger domains, is diminished in multiple cancers and possesses tumor-suppressor features such as inhibition of cell proliferation mediated by KRAS [37]. In this study, we also showed that KLF2 can function as tumor suppressor and its' expression could be suppressed by LINC01133 in NSCLC cells. Meanwhile, LINC01133 also silenced E-cadheirn in NSCLC cells, and loss of E-cadherin is an important hallmark of epithelial-mesenchymal transition (EMT) process. Numerous of evidence have revealed that EMT is implicated in the promotion of tumor cell invasion and metastasis, and could be a potent mechanism for promoting the detachment of cancer cells from primary tumors [38, 39]. Our data suggest that LINC01133 involved in NSCLC cells migration and invasion might partly through affecting EMT process by repressing E-cadherin expression in NSCLC cells.

In conclusion, we have shown for the first time that LINC0133 expression was up-regulated in NSCLC tissues, suggesting that its up-regulation may be a negative prognostic factor for NSCLC patients. Knockdown of LINC01133 exerted tumor-suppressive functions by reducing cell proliferation, migration and invasion as well as inducing apoptosis in NSCLC cells. Furthermore, LINC01133 mediated the oncogenic effects is partially through its epigenetic silencing of the KLF2, P21 and E-cadherin expression by interacting with EZH2 and LSD1. Our findings further the understanding of NSCLC pathogenesis, and facilitate the development of lncRNA-directed diagnostics and therapeutics against this disease. However, other possible targets and mechanism that underlie regulatory behaviors were not investigated in our study, which still remains to be fully understood and needs to be further investigated.

## MATERIALS AND METHODS

### NSCLC samples collection

68 paired NSCLC and adjacent non-tumor tissues were collected from patients who underwent surgery at Jiangsu Province Hospital between 2010 and 2011, and were diagnosed with NSCLC based on histopathological evaluation. Clinicopathological characteristics, including tumor-node-metastasis (TNM) staging were recorded. There was no local or systemic treatment in these patients before surgery. All collected tissue samples were immediately snap-frozen in liquid nitrogen and stored at −80°C until required. Our study was approved by the Research Ethics Committee of Nanjing Medical University, China. Written informed consent was obtained from all patients.

### Cell lines

Five NSCLC adenocarcinoma cell lines (PC9, SPC-A1, NCI-H1975, H1299, and A549), and three NSCLC squamous carcinomas cell lines (H520, H1703, and SK-MES-1) were purchased from the Institute of Biochemistry and Cell Biology of the Chinese Academy of Sciences (Shanghai, China). A549, H1975, H1299, H1703 and H520 cells were cultured in RPMI 1640; 16HBE, SK-MES-1, PC9 and SPC-A1 cells were cultured in DMEM (GIBCO-BRL) medium supplemented with 10% fetal bovine serum (FBS), 100 U/ml penicillin and 100 mg/ml streptomycin (Invitrogen, Carlsbad, CA, USA) at 37°C/5% CO_2_.

### RNA extraction and qPCR assays

Total RNA was isolated with Trizol reagent (Invitrogen) according to the manufacturer's instructions. Total RNA (1ug) was reverse transcribed in a final volume of 20 μl under standard conditions for the PrimeScript RT reagent Kit (TaKaRa, Dalian, China). SYBR Premix Ex Taq (TaKaRa, Dalian, China) was used to determine LINC01133 and targets expression levels, following the manufacturer's instructions. Results were normalized to the expression of glyceraldehyde-3-phosphate dehydrogenase (*GAPDH*). The specific primers are shown in Supplementary Table S1.

### Cell transfection

Human LINC01133 cDNA and short-hairpin RNA directed against LINC01133was ligated into the pCDNA3.1 and pENTR™ H1 vector. Plasmid vectors (pCDNA-LINC01133, pCDNA-KLF2, sh-LINC01133 and empty vector) for transfection were prepared using DNA Midiprep or Midiprep kits (Qiagen, Hilden, Germany), and transfected into SPC-A1, A549, PC9 or H1975 cells. The si-LINC01133 or si-NC were transfected into A549, PC9 or H1975 cells. A549, PC9 or H1975 cells were grown on six-well plates to confluency and transfected using Lipofectamine 2000 (Invitrogen) according to the manufacturer's instructions. At 48 h post-transfection, cells were harvested for qPCR or western blot analysis.

### Cell proliferation assay

Cell proliferation was monitored using a Cell Proliferation Reagent Kit I (MTT) (Roche Applied Science) and EdU assay kit (Life Technologies Corporation Carlsbad, CA, USA). For MTT assays, cells were seeded into a 96-well plate. Cells per well were added 20 μl MTT solution. Plates were incubated for 6 h, and then the absorbance at 490 nm was measured. For EdU incorporation assay, cells were cultured in 24-well plates. 10 μM of EdU was added to each well and cells were cultured for an additional 2 h. Then the cells were fixed with 4% formaldehyde for 30 min. After washing, EdU can be detected with a Click-iTR EdU Kit for 30 min, and the cells were stained with DAPI for 10 min and visualized using a fluorescent microscope (Olympus, Tokyo, Japan). The EdU incorporation rate was expressed as the ratio of EdU positive cells to total DAPI positive cells (blue cells), and were counted using Image-Pro Plus (IPP) 6.0 software (Media Cybernetics, Bethesda, MD, USA).

### Flow cytometry

Cells were harvested 48 hr after transfection by trypsinization, and double stained with FITC-Annexin V and Propidium iodide (PI) using the FITC Annexin V Apoptosis Detection Kit (BD Biosciences). Then, the cells were analyzed with a flow cytometry (FACScan® BD Biosciences) equipped with a CellQuest software (BD Biosciences). Cells for cell cycle analysis were stained with PI using the CycleTEST™ PLUS DNA Reagent Kit (BD Biosciences) following the protocol and analyzed by FACScan. The percentage of the cells in G0/G1, S, and G2/M phase were counted and compared.

### Cell migration and invasion assays

For cell migration and invasion assays 24-well transwell chambers with 8 μm pore size polycarbonate membrane was used (Corning Incorporated, Corning, NY, USA). Cells were seeded on the top side of the membrane pre-coated with Matrigel (BD, Franklin Lakes, NJ, USA) (without Matrigel for cell migration assay). Incubation for 24h, cells inside the upper chamber were removed with cottons swabs, while cells on the lower membrane surface were fixed and then stained with 0.5% Crystal violet solution. Five randomly fields were counted randomly in each well.

### *In vivo* tumor formation assay

Four weeks female athymic BALB/c nude mice were maintained under pathogen-free conditions and manipulated according to protocols approved by the Shanghai Medical Experimental Animal Care Commission. A549 cells were stably transfected with sh-LINC01133 and empty vector and harvested, washed with phosphate-buffered saline (PBS) and re-suspended at a concentration of 1 × 10^8^ cells/ml. A total of 100 μL of suspended cells was subcutaneously injected into each mouse. Tumor growth was examined every 3 days, and tumor volumes were calculated using the equation volume=length × width2/2. At 18 days post-injection, mice were euthanized, and the subcutaneous growth of each tumor was examined. The protocol was approved by the Committee on the Ethics of Animal Experiments of the Nanjing medical University.

### RNA immunoprecipitation

For immunoprecipitation (IP) of endogenous PRC2 and LSD1 complexes from whole-cell extracts, cells were lysed. The supernatants were incubated with protein A/G Sepharose beads coated with antibodies that recognized EZH2, SUZ12, LSD1, SNRNP70 or with control IgG (millipore) for 6hr at 4°C. After the beads were washed with wash buffer, the complexes were incubated with 0.1% SDS/0.5 mg/ml Proteinase K (30 min at 55°C) to remove proteins, respectively. The isolated from the IP materials was further assessed by qPCR analysis.

### RNA pull-down assays

LINC01133 RNAs were in vitro transcribed using T7 RNA polymerase (Ambio life), which was then purified using the RNeasy Plus Mini Kit (Qiagen) and treated with RNase-free DNase I (Qiagen). Transcribed RNAs were biotin-labeled with the Biotin RNA Labeling Mix (Ambio life). Positive, negative and Biotinylated RNAs were mixed and incubated with A549 cell lysates. Magnetic beads were added to each binding reaction, followed by incubation at room temperature. Then, the beads were washed with washing buffer. The eluted proteins were detected by Western blot analysis.

### Chromatin Immunoprecipitation

A549 and PC9 cells were treated with formaldehyde and incubated for 10 mins to generate DNA-protein cross-links. Cell lysates were then sonicated to generate chromatin fragments of 200-300 bp and immunoprecipitated with LSD1, H3K4me2, EZH2 and H3K27me3-specific antibody (Millipore) or IgG as control. Precipitated chromatin DNA was recovered and analyzed by qPCR.

### Western blot assay and antibodies

Cells protein lysates were separated by 10% SDS-polyacrylamide gel electrophoresis (SDS-PAGE), transferred to 0.22μm NC membranes (Sigma) and incubated with specific antibodies. ECL chromogenic substrate was used to were quantified by densitometry (Quantity One software; Bio-Rad). GAPDH antibody was used as control, Anti-GAPDH, P21, CDK2, CDK4, CDK6, cyclinD1, cyclinD3, PARP, cleaved PARP and E-cadherin (1:1000) were purchased from Cell Signaling Technology, Inc (CST); Anti-KLF2 were purchased from sigma.

### Statistical analysis

All statistical analyses were performed using SPSS 17.0 software (IBM, Chicago, IL, USA). The significance of differences between groups was estimated by the Student t-test, Wilcoxon test or χ2 test. DFS and OS rates were calculated by the Kaplan–Meier method with the log-rank test applied for comparison. Pearson correlation analyses were used to investigate the correlation between LINC01133 and KLF2, P21 or E-cadherin expressions. Two-sided p-values were calculated, and a probability level of 0.05 was chosen for statistical significance.

## SUPPLEMENTARY FIGURES AND TABLE




