# Supplementary material for: Long non-coding RNA LINC01133 represses KLF2, P21 and E-cadherin transcription through binding with EZH2, LSD1 in non small cell lung cancer
Source: Oncotarget. 2016 Jan 30;7(10):11696–707. doi: 10.18632/oncotarget.7077 (PMC4905504; doi:10.18632/oncotarget.7077)
Supplement: Supplementary file 1 [file oncotarget-07-11696-s001.pdf]

SUPPLEMENTARY FIGURES AND TABLE

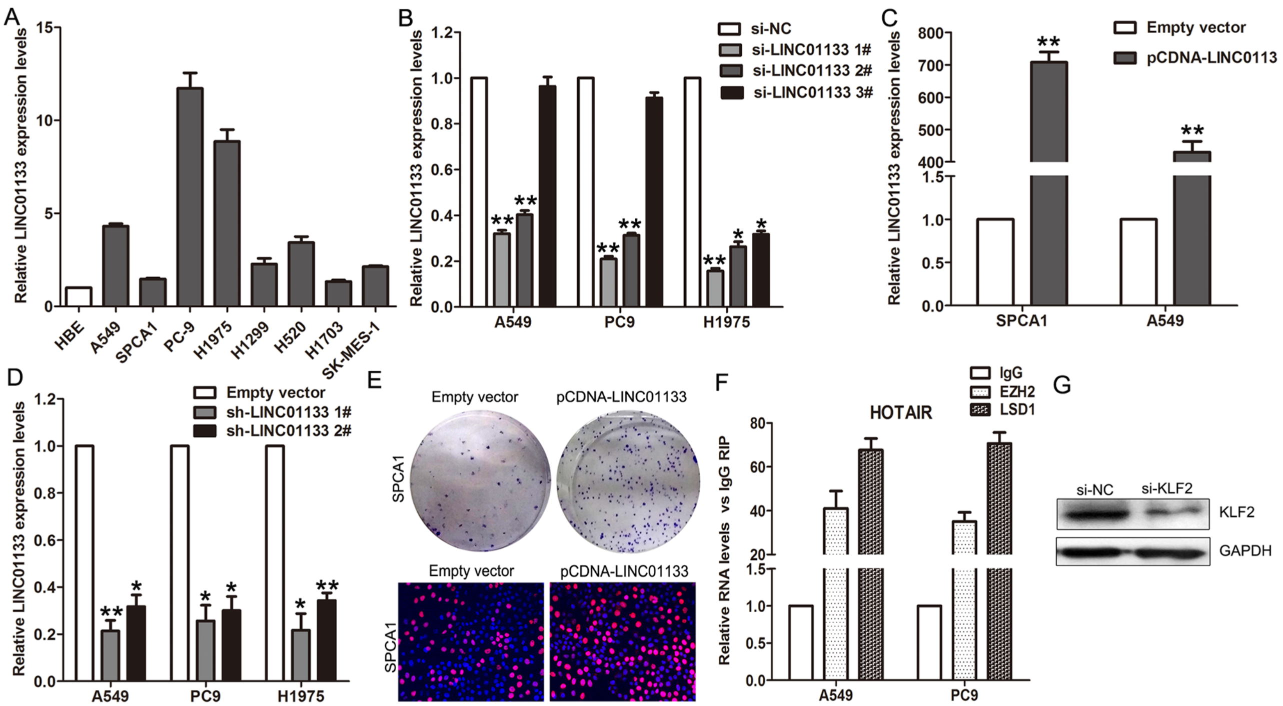

Supplementary Figure S1: A–D. LINC01133 expression levels were detected by qPCR in NSCLC cells. E. EdU incorporation and colon formation assays to evaluate the effect of LINC01133 over-expression on cell proliferation of SPCA1. **F.** RNA levels in immunoprecipitates were determined by qPCR. Expression levels of HOTAIR RNA were presented as fold enrichment relative to IgG immunoprecipitates. **G.** Protein level of KLF2 was determined by western blot. $^{*}P < 0.05$ and $^{**}P < 0.01$.

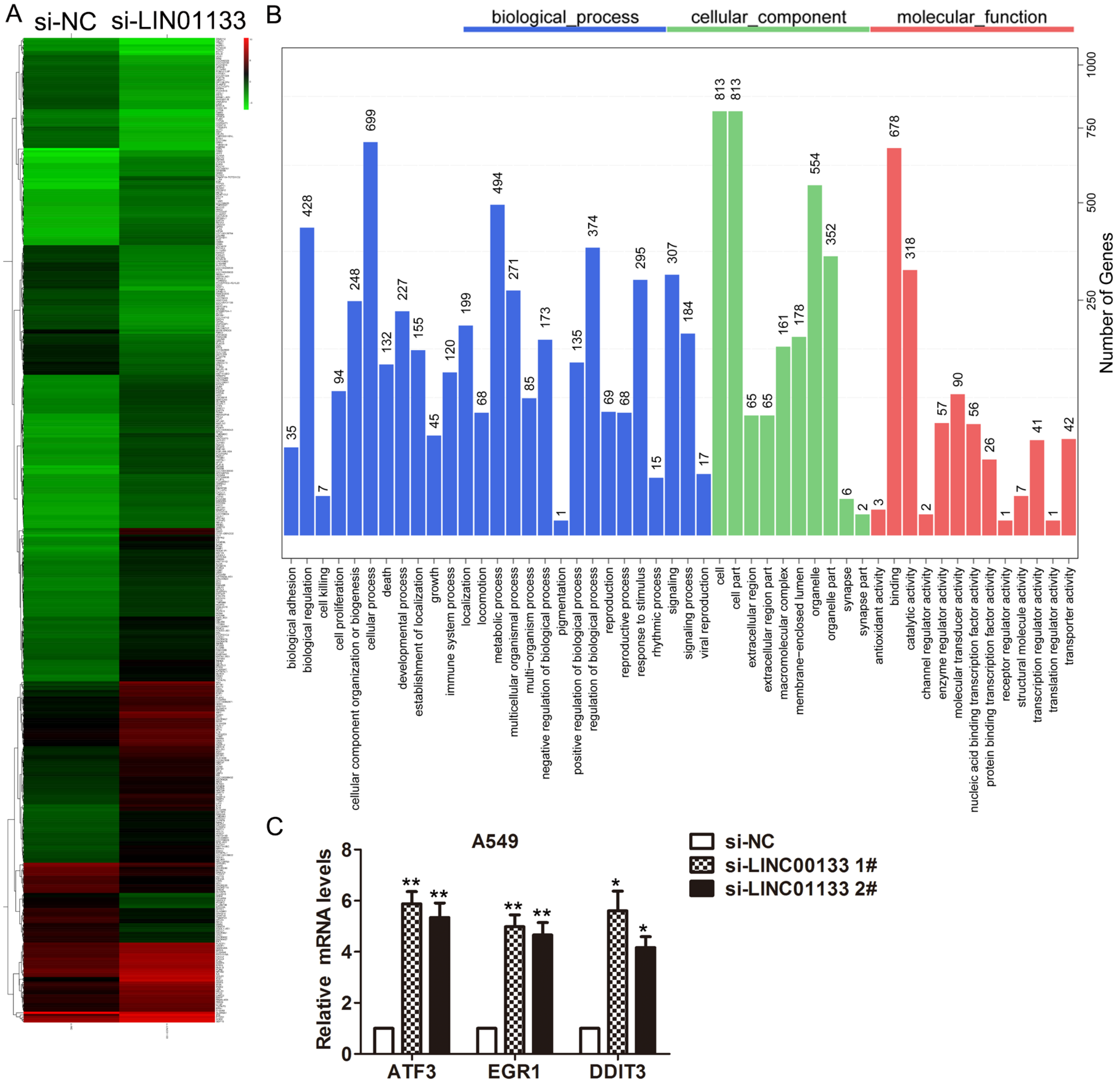

Supplementary Figure S2: Identification of LINC01133 targets in NSCLC cells. A. The entire repertoire of LINC01133-affected genes was investigated by RNA sequencing in A549 cells after knockdown of LINC01133. **B.** GO and pathway analysis of the up or down-regulated genes after LINC01133knockdown. **C.** The ATF3, DDIT3 and EGR1 expression levels were detected by qPCR. $^{*}P < 0.05$ and $^{**}P < 0.01$.

Supplementary Table S1: The primers, siRNAs and shRNAs sequence
See Supplementery File 1
